# Femicide and murdered women’s children: which future for these children orphans of a living parent?

**DOI:** 10.1186/s13052-015-0173-z

**Published:** 2015-09-29

**Authors:** Pietro Ferrara, Olga Caporale, Costanza Cutrona, Annamaria Sbordone, Maria Amato, Giulia Spina, Francesca Ianniello, Giovanna Carmela Fabrizio, Chiara Guadagno, Maria Cristina Basile, Francesco Miconi, Giacomo Perrone, Riccardo Riccardi, Alberto Verrotti, Massimo Pettoello-Mantovani, Alberto Villani, Giovanni Corsello, Giovanni Scambia

**Affiliations:** Institute of Pediatrics, Catholic University of Sacred Heart, Rome, Italy; Campus Bio-Medico University, Rome, Italy; Department of Pediatrics, University of Perugia, Perugia, Italy; Institute of Pediatrics and Residency program, University of Foggia, Foggia, Italy; Pediatric and Infectious Disease Unit, Bambino Gesù Children’s Hospital, IRCCS, Rome, Italy; Department of Sciences for Health Promotion and Mother and Child Care, University of Palermo, Palermo, Italy; Department of Obstetrics and Gynecology, Catholic University of Sacred Heart, Rome, Italy

**Keywords:** Femicide, Intimate partner, Children, Treatment, Psychological

## Abstract

**Background:**

To assess the prevalence of femicides in Italy over the last three years and the potential long lasting effects of these traumatic events for the children of a woman who dies a violent death.

**Methods:**

The data used in this study come from an internet search for the number of femicides occurring in Italy between 1^st^ January, 2012 and 31^st^ October, 2014.

**Results:**

The total number of femicides was 319; the average age of murdered women was 47.50 ± 19.26. Cold arms in the form of sharp object -mostly knives- have caused the death of 102/319 women; firearms were used in 87/319 cases; asphyxiation was the chosen method in 52/319 cases. About the place where the femicides occurred, 209/319 were committed inside the victim’s house. Children of women who died a violent death were 417 with a total of 180 minors in less than three years. A total of 52/417 children were witness to the killing and, among these 30/52 were minors; in 18/417 cases, children were murdered together with their mother and among these 9/18 were minors.

**Conclusions:**

Long-term studies are needed to ascertain what happens to these children, to understand what are the most appropriate psychological treatments, the best decisions about the contact with their father and the best placement for these children.

## Introduction

Homicide is an important cause of premature mortality worldwide [1]. It caused the deaths of almost half a million people (437,000) across the world in 2012 [2]. The global homicide rate is almost four times higher for male (homicides) than for female homicides. However, the pattern is different when looking at intimate partner homicide [1]. Women are more likely to be murdered by intimate-partners: almost half of all female homicides were perpetrated by an intimate partner or family members in 2012, compared to less than 6 per cent of male homicides [2].

In 1992, femicide was defined as “the killing of women committed by a man by virtue of the fact that they are women, emphasizing the social aspects of the violent act” [3], but broader definitions include any killings of women or girls [4].

According to 2013 UNODC (United Nations Office on Drugs and Crime) homicide statistics, the murder of women is less common in Europe than in Asia, Africa and Americas. Moreover, the prevalence of Intimate Partner Femicide (IPF) is lower in Italy than in any other European Country [2].

Physical violence against the victim is the primary risk factor for IPF [5]. Also actual or imminent separation, abuser’s access to a firearm, prior threats with a weapon, prior threats to kill the victim, a stepchild in the household, problematic alcohol and illicit drug use and mental health problems are associated with substantially higher risk for femicide [5–8]. The only sociodemographic risk factor for intimate partner femicide is the abuser’s lack of employment [5].

The intimate partner homicide often involve the murder of family members or bystanders, such as the couple’s children, relatives or new partners of the victims and have long-term consequences on remaining family members [1].

Little is known about the number of orphaned children who have instantaneously lost both parents (one from death, the other from incarceration or suicide).

The aim of this study is to assess the prevalence of femicides in Italy over the last three years. Although in the last years there has been a growing research literature concerning the problem of femicide, to date there are no studies in literature that focused specifically on children of the murdered women. Even when publications concerning femicide include some information on children, the data are always limited to the description of few isolated cases [9]. We focused for the first time on this important, usually ignored, aspect. Potential long lasting effects of these traumatic events for the children of a woman who dies a violent death are also discussed.

## Methods

The data used in this study come from an internet search for the number of femicides occurring in Italy between 1^st^ January, 2012 and 31^st^ October, 2014. Newspaper indexes, news websites and internet search engines such as Google were used. The search keywords included: femicide, intimate partner, homicide, children and parental killing.

All women’s violent deaths occurred in Italy were included.

At the level of the victim, we explored the victim’s age, race/ethnicity, relationship with the perpetrator (husband, boyfriend, ex-husband/boyfriend, other) and whether she was a sex worker or known to be pregnant at the time of death. Homicide characteristics studied include weapon type causing the fatal injury, the underlying cause, whether homicide took place in the home and status of perpetrator after homicide (alive or committed suicide).

We also looked at the presence of children in the household or family, their relationship with the perpetrator (father, step-father, other) and whether they witnessed the murder, have been killed with their mother or were orphaned by this act.

The study was carried out in compliance with the Helsinki Declaration.

## Results

In Italy, from 1^st^ January, 2012 to 31^st^ October, 2014the total number of femicides was 319; a woman was murdered every three days in the last three years in Italy. Considering the yearly number, in 2012 a total of 126 women were murdered, 134 femicides were committed in 2013 and finally 59 in 2014. The average age of murdered women was 47.50 ± 19.26; the age distribution into bands and nationality of victims are shown in Table 1. The number of prostitutes among murdered women was 28/319 (8.77 %). Considering the femicides territorial distribution in Italy, we found that the major number of episodes occurred in northern Italy: 64/126 (50.79 %) in 2012, 45/134 (33.58 %) in 2013 and 28/59 (47.46 %) in 2014; while the remaining cases were almost equally distributed among central, southern Italy and Italian islands (Sardinia and Sicily).

**Table 1:** Characteristics of IPF (n = 319) in Italy, 2012-2014

Characteristic	n	(%)
Age (yrs)		
<18	3	0.94 %
18-25	34	10.66 %
26-35	61	19.12 %
36-45	59	18.50 %
46-60	74	23.20 %
61-75	44	13.79 %
>75	39	12.4 %
Unknown	5	1.57 %
Country		
Italy	228	71.47 %
Eastern Europe	35	11 %
Latin America	18	5.64 %
Europe	14	4.38 %
Africa	12	3.76 %
Other	8	2.50 %
Unknown	4	1.25 %

Cold arms in the form of sharp object -mostly knives- have caused the death of 102/319 (32 %) women; firearms were used in 87/319 (27.27 %) cases; asphyxiation was the chosen method in 52/319 (16.30 %) cases.

The femicides were committed by a current or former intimate partner in 197/319 (61.75 %) cases; the author was a woman’s family member in 46/319 (14.42 %) cases. Only in few cases, the woman didn’t know the murderer: none in 2012, 2/134 (1.49 %) in 2013, 2/59 (3.39 %) in 2014.

About the place where the femicides occurred, 209/319 (65.51 %) were committed inside the victim’s house. Table 2 illustrates the characteristics of IPF and the circumstances surrounding the femicide. Some of the women murdered were pregnant at the time of the death: 2/126 (1.59 %) in 2012, 1/134 (0.75 %) in 2013 and 1/59 (1.69 %) in 2014.

**Table 2:** Characteristics of IPF (n = 319) in Italy, 2012-2014

Characteristic	n	(%)
Killing method		
Sharp object	102	32 %
Firearm	87	27.27 %
Asphyxiation	52	16.30 %
Improper weapon	28	8.78 %
Punch	19	5.96 %
Other	21	6.58 %
Unknown	10	3.13 %
Location		
Victim’s home	209	65.51 %
Other public place	52	16.30 %
Victim’s workplace	18	5.64 %
Victim’s car	16	5.02 %
Park	6	1.88 %
Other	8	2.50 %
Unknown	10	3.2 %
Relationship with the perpetrator		
Current intimate partner	143	44.83 %
Former intimate partner	54	16.93 %
Family member	46	14.42 %
Friend	15	4.70 %
Colleague	13	4.08 %
Client of sex workers	13	4.08 %
No relationship	4	1.25 %
Unknown	31	9.72 %
Circumstances		
End of relationship	66	20.69 %
Jealousy	53	16.61 %
Quarrel	27	8.46 %
Economic problems	26	8.15 %
Men’s psychiatric disorder	23	7.21 %
Woman’s illness	17	5.33 %
History of violence	11	3.45 %
Prostitutions	10	3.13 %
Unrequited love	5	1.57 %
Work-related quarrel	3	0.94 %
Attempted sexual assault	1	0.31 %
Other	21	6.58 %
Unknown	56	17.55 %

Children of women who died a violent death were 153 (63 % male, 37 % female) in 2012, 173 (54 % male, 46 % female) in 2013 and 91 (58 % male, 42 % female) in 2014, for a total of 417 children in less than three years. Among these children, there were 79/153 (51.63 %) minors (age <18 years) in 2012, 52/173 (30.06 %) minors in 2013 and 49/91 (53.85 %) in 2014, for a total of 180 minors in less than three years.

A total of 52/417 (12.47 %) children were witness to the killing and, among these, 30/52 (57.69 %) were minors. In some cases, also children were murdered together with their mother: 18/417 (4.32 %) and, among these, 9/18 (50 %) were minors. The murder was committed by the father of the children in 123/319 (38.56 %) cases of femicide. Children whose father dead by his own hand after killing their mother were 49/417 (11.75 %) and, among these orphans, 21/49 (42.86 %) were minors.

Information about where and with whom the minors will live after their mother’s murder were really few. We found these information only for some sparse cases: minors were placed with maternal grandparents in 29 cases; only in 1 case the child was placed with paternal grandparents. In another case, maternal grandparents were elderly and poor to take care of the child, so that he was placed in a foster-home. We also found that in 3 cases the children were placed with their aunts and uncles. We found that in 8 cases children continued to live with their father. Four sisters in 2012 were already been placed with another family, before their mother’s murder, because they had experienced violence from their father (who then killed their mother); likewise, 2 brothers in 2013 were already been placed with another family, before their mother’s death, because she was an alcoholic.

## Discussion

**Fig. 1:**
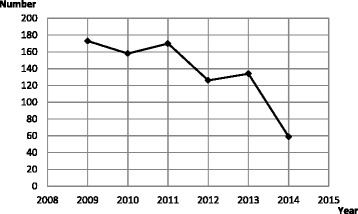
Cumulative number of femicides per year

Very few studies are available about incidence of femicides in Italy. According to EURES-ANSA research performed in 2012, between 2000 and 2011, a total of 2061 femicides were recorded in Italy, so that the yearly number of total femicides averaged at about 171.7 cases. Moreover, 501 femicides were committed in Italy between 2009 and 2011, with a mean number of 167 femicides per year [10]. Our findings for the years 2012–2014 show a reduction of this trend (Fig. 1). The present results revealed that the total number of femicides was significantly decreased in 2014. A potential explanation could be that data were collected until October 31^st^; however, considering the average number per month, downward trend is confirmed: 10.5 women per month in 2012, 11.2 in 2013 and 5.5 in 2014. We hypothesized that this drastic reduction could be due to a new innovative law enacted by the Italian Government recently: “Law n° 119–15.10.2013”. This law contains new rules to fight gender-based violence in order to prevent femicide and protect victims. Moreover, the Convention of Istanbul was ratified in Italy on 19 June 2013 to prevent violence against women, protect female victims and enable criminal prosecution of offenders; however signatures from two other States of the European Union are necessary to make the document effective [11]. Further research to understand the underlying causes of this trend-reduction is needed.

Moreover in a recent Italian study, the analysis of 40 years of mortality shows only a slight decrease of the murders of women nationwide. The decrease has taken place mainly in the South and Islands, thus at the end of the observation period the North-West assumes a greater weight than the South and Islands; so the Authors cannot exclude that part of the decrease in murders of women can be attributed to the overall decrease in homicides related to criminal activity [12]. Besides in recent years there is much debate over the issue and there is more attention from everyone: the media, law enforcement agencies, police force, institutions, citizens and just this awareness-raising may discourage femicides.

Our data also confirm prior findings that the rate of femicide in Italy is lower than in other countries in the world: according to the Spanish Centre “Reina Sofia” research, in 2003 among European countries, the UK, Spain, Germany, Austria and Denmark report higher femicide rates than Italy [13].

Previous research performed by Moracco and colleagues revealed that younger women are at greater risk of partner homicide [7]. Our results are consistent with these findings: 72 % of the victims are ≤ 60 years old. The rates were highest for women in the 46 to 60 age group, followed by women in the 26–35 age group.

The present results show that the victims in 71.47 % cases were Italian, suggesting that socio-demographic characteristics are not significantly predictive of femicide risk. Likewise, the findings that the major number of femicides occurred in northern Italy take us to the same conclusion.

We found that current and former intimate partners were the perpetrators in more than half of the homicide deaths of women in Italy. Our data also confirm prior findings that actual or imminent separation appears to be a high-risk situation, since, in our data, the end of the relationship results the most frequent motive behind the femicide [14, 15]. Consistent with previous researches, women who separated from their abusive partners after cohabitation experienced increased risk of femicide, particularly when the abuser was left by his partner for a different partner [16].

The most chosen method of killing was through sharp object, mostly knives, followed by firearms and then by asphyxiation. Gun availability still increased homicide risks: this is probably due to gun-owning abusers’ much greater likelihood of using a gun in the worst incident of abuse [17, 18]. Besides, regarding the method of asphyxiation, our data are similar to those of research performed in India, South Africa, and Finland, where 10–20 % of femicides were carried out by this killing method [18–20].

Children whose father kills their mother are orphaned by this act: they not only lose their mother, but also their father to prison or suicide. We found that, in the last three years, 180 children aged <18 years were deprived of their mother by the catastrophe of her death. Overall 90 % of all femicides reported by us did not have information about placement, access and custody of children after the killing.

Placement after the death is very problematic. The caregivers often exert pressure on the child to forget what happened by not speaking about it, negating the child’s version of events, and responding with silence and evasion to the child’s questions so that the children will have difficulty to effectively mourn their losses [21]. This suppression is particularly likely if his carers are kin [9].

As attention is focused on the victim and on the perpetrator of the crime, the couple’s children become the neglected victims.

Of all the traumatic events that children can experience, none can be more horrific than witnessing the murder of one parent by another. Besides, children who witness parental homicides sometimes were left alone with the dead body of their mother, may have had to find help or attempt to defend her. Some of them were also witness to their father’s subsequent suicide and may be the only source of information for the police and the social services.

Many studies have described regressive and maladaptive responses that accompany these traumatic events. These disorders include enuresis, encopresis, sleep disturbance, temper tantrums, flashbacks, dissociation, anxiety and psychosomatic disorders, and passive and aggressive behaviors [22]. According to research performed by Black and colleagues, most of the children present at the killing had symptoms of post-traumatic stress disorder, whereas it was not found in those who were absent from home at the time of the killing [8]. Moreover, several studies have concurred that violence observed by children has a high probability of being reenacted later in life [23].

Unfortunately there are no data about locations and people whom the minor will live after their mother’s murder and this is the first manuscript in Italy which seeks to raise awareness on the issue. The children of mothers killed usually are entrusted with relatives or placed in foster homes and, in extreme cases, in foster families. Anyway the removal from the biological family causes devastating consequences both psychological and physical.

If treatment is neglected or postponed, adaptations to satisfactory and optimal functioning, both transitory and prolonged, can be severely compromised. Immediate and intensive care for these children and their family is essential [24].

Black and colleagues, as a result of their experience with these troubled children, suggested some recommendations that include: immediately after the killing, children’s placement should be with familiar people; primary health care services should be alerted about the children and early consultations sought from child mental health services; expert advice should be sought regarding the possibility of attending their mother’s funeral and of visiting their father [9]. In the longer term, permanent placement planning should begin as soon as possible: the emergency placement should not become permanent by default; therapeutic help should be available as needed, especially for children who witnessed the killing.

Improved ability to identify women who are at risk for IPF may facilitate prevention and enable more appropriate allocation of resources. When women are identified as abused in medical settings, it is important to assess perpetrators’ access to guns or another weapon and to warn women of the risk weapon present. If a woman is planning to leave her abuser, it is critical to advice her not to confront him personally with her decision; instead, she needs to leave when he is not present and leave a note or call him later. Different levels of prevention are possible and include strategies directed at risk factors for homicide in general; for example, many analysis suggest that increasing employment opportunities, preventing substance abuse, and restricting abusers’ access to guns can potentially reduce rates of IPF [5].

## Conclusions

Few and sparse attention has been focused on children whose mother was murdered. For child psychiatry teams these are difficult cases and few individuals have experience of working with such children in their professional lifetime. Where children should live and with whom, whether they should attend the funeral, see their father in prison are questions that require consideration and discussion. Judges, police, social workers, or offices that attend the victims could make decisions about protection, on the basis of empirical data and not merely using intuitive criteria. For this reason, long-term studies are needed to ascertain what happens to these children (especially when they grow up), to understand what are the most appropriate psychological treatments, the best decisions about the contact with their father (when he is the murderer) and the best placement for these children.
